# Lung Metastasis of Primary Alveolar Soft-Part Sarcoma Occurring 20 Years after Initial Treatment

**DOI:** 10.1155/2013/690520

**Published:** 2013-11-27

**Authors:** R. F. Falkenstern-Ge, M. Kimmich, M. Wohlleber, A. Grabner, G. Friedel, G. Ott, I. Leuschner, M. Kohlhäufl

**Affiliations:** ^1^Division of Pulmonology, Klinik Schillerhoehe, Center for Pulmonology and Thoracic Surgery, Teaching Hospital of the University of Tuebingen, Solitude Street 18, Gerlingen, 70839 Stuttgart, Germany; ^2^Department of Clinical Pathology, Robert Bosch Krankenhaus, Teaching Hospital of the University of Tuebingen, Auerbachstrasse 110, 70376 Stuttgart, Germany; ^3^Division of Thoracic Surgery, Klinik Schillerhoehe, Center for Pulmonology and Thoracic Surgery, Teaching Hospital of the University of Tuebingen, Solitude Street 18, Gerlingen, 70839 Stuttgart, Germany; ^4^Department of Pediatric Pathology, Institute of Pathology, Kiel, Germany

## Abstract

A 30-year old woman was referred to our center because of suspicion of a primary lung tumor of the right upper lobe. Histological examination of the lung lesion revealed lung metastasis of a previously treated alveolar soft part sarcoma of the musculus vastus medialis of the right femur, which was resected 20 years ago. Alveolar soft-part sarcoma is a rare malignant tumor that occurs most often in the soft tissue of lower limbs. It is a slow-growing malignant soft tissue tumor arising in muscle tissue, usually in young adults. Due to pleural and extensive mediastinal infiltration with bilateral lung metastases, a systemic treatment with chemotherapy doxorubicin and ifosfamide was initiated. Late metastases from previously treated alveolar part sarcoma should be considered in patients with suspicious lung lesions even if surgical treatment was performed a long time ago.

## 1. Introduction

Alveolar soft-part sarcoma is a rare malignant tumor that occurs most often in the soft tissue of lower limbs. Alveolar soft-part sarcoma is a very uncommon soft tissue tumor known for late metastases to lung, bone, and brain. Lillehei and coworkers reported an interval of 21 years between primary presentation and development of lung metastasis and 33 years between primary presentation and development of brain and renal masses. Our case represents the second longest interval of 20 years between primary tumor presentation and development of a pulmonary recurrence.

## 2. Background

**Figure 1:**
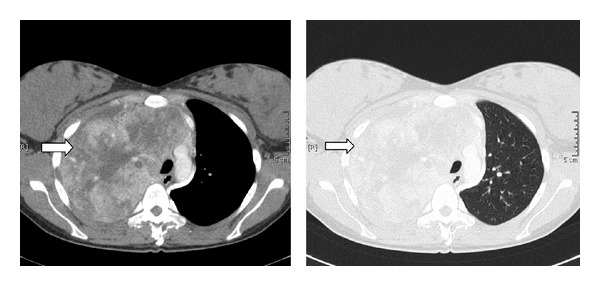
Contrast-enhanced computed tomography scan of a large tumor mass in the right upper lobe with extensive mediastinal infiltration.

A 30-year old female patient was referred to our institution because of a large lung mass of the right upper lobe. The patient complained about increasing dyspnea for 2 months. Her initial physical examination was unremarkable and laboratory tests were all within normal limits. Spirometry revealed a mild restrictive pattern (TLC 88% soll) and reduced diffusion capacity (DLCO-SB 52% soll). CT thorax scan (Figure 1) revealed a huge tumor mass 13.2 × 13.7 × 11 cm in diameter of the right upper lobe of the lung with extensive pleural infiltration and infiltration of the mediastinum.

**Figure 2:**
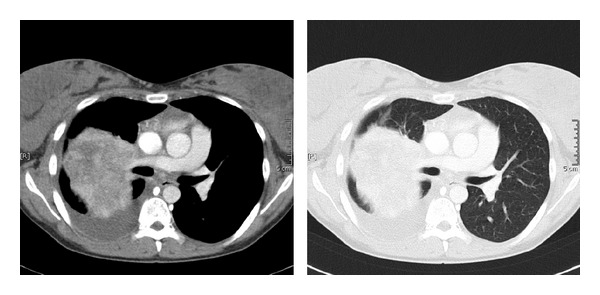
Contrast-enhanced computed tomography revealed pleural infiltration on the right side.

Contrast-enhanced computed tomography scan (Figure 2) also revealed ipsilateral right-sided pleural tumor infiltration.

The patient, interestingly, had a history of alveolar soft-part sarcoma of the musculus vastus medialis of the right femur successfully resected 20 years ago. The primary alveolar soft-part sarcoma had been initially staged as IA (T1 N0 M0) 20 years ago. All regular follow-up evaluations were unremarkable. However, the last chest X-ray evaluation was performed 8 years ago. Clinical workup revealed bilateral pulmonary metastases with ipsilateral pleural infiltration.

**Figure 3:**
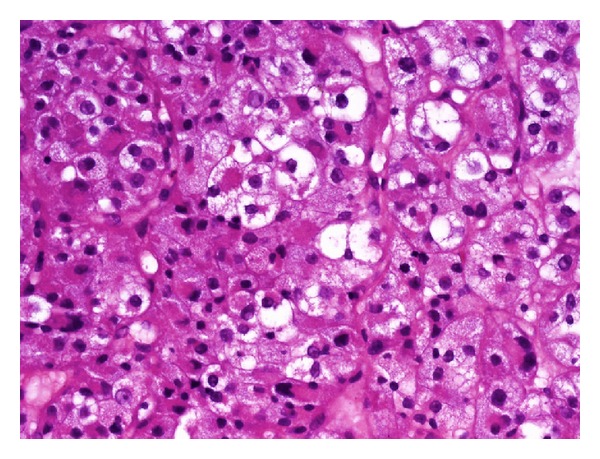
The cytoplasm of the tumor cells was broad and clear or eosinophilic. The nuclei were round and uniform, frequently with a single nucleolus.

Histological workup of material obtained during bronchoscopy showed a solid tumor composed of medium-sized to large cells arranged in compartmentalizing cords and nests, which were separated by delicate septa with capillaries. The cytoplasm of the tumor cells was broad and clear or eosinophilic. The nuclei were round and uniform, frequently with a single nucleolus (Figure 3).

After immunohistochemistry demonstrating reactivity of the tumor cells for protein S100, but not for melanocytic markers or keratin markers, and comparison with the primary tumor, a diagnosis of metastatic alveolar soft-part sarcoma was rendered.

## 3. Discussion 

Alveolar soft-part sarcoma was initially described in 1952 by Christopherson et al. [1]. It is a rare tumor which accounts for less than one percent of soft tissue sarcomas [2–4]. There is a female predominance and the median age at diagnosis is 22 years for females and 27 years for males [4], but the tumor can also occur in children as young as 2 years old. The most common site of origin is the lower limb, followed by the trunk and the upper limbs. In children, the tumor is not infrequently found in the tongue and orbit. However, the tumor can arise also in uncommon localizations such as stomach, liver, or breast [2–4]. As in our case, the granular tumor cells are often arranged in cords and nests (“alveoli”) separated by connective tissue septa that have a rich vascular network, as in our case [4].

Pulmonary metastases are encountered in 42–65% of patients, and the lung is most commonly involved. The brain and the skeleton are the two next most commonly involved metastatic sites [3]. At first presentation, up to 24% of patients show metastatic disease. Of those patients without metastases at presentation, 40% of patients will develop metastatic disease within five years and 62% of those patients within 10 years of followup [4]. Because of the high prevalence of pulmonary metastasis and good clinical results of pulmonary metastasectomy, annual screening of chest CT for an indefinite period has been advocated for patients with alveolar soft-part sarcoma [5]. The primary treatment option after initial staging should be complete surgical resection. Complete resection of the primary, as well as of the recurring, tumor manifestations is essential for recurrence control and long-term survival [6, 7].

Chemotherapy is widely used in the treatment of nonresectable advanced disease, primarily with palliative intention. Initial standard chemotherapy for advanced or metastatic soft tissue sarcoma consists of single-agent anthracycline (mainly doxorubicin) or an anthracycline-based combination with, for example, ifosfamide and dacarbazine. The use of doxorubicin is limited, due to its cumulative cardiac toxicity [8]. Regimens containing doxorubicin, cyclophosphamide, cisplatin, vincristine, dacarbazine, and other agents have not been shown to be effective as preoperative and/or postoperative treatment of alveolar soft-part sarcoma [8]. Gemcitabine and docetaxel are another frequently used combination as a second-line regimen in alveolar soft-part sarcoma [8–10]. It is impossible to draw definitive conclusions about the effectiveness of adjuvant radiotherapy for alveolar soft-part sarcoma based on the limited number of patients in published series. However, several studies have suggested that adjuvant radiotherapy is beneficial in this setting. Sherman et al. reported prolonged (24–150 months) local control and recurrence control in all of six patients who underwent surgery and adjuvant radiotherapy (56–65 Gy) [11].

Our patient had been diagnosed with alveolar soft-part sarcoma of the lower extremity 20 years ago and was initially been successfully treated with complete resection of the tumor and adjuvant radiochemotherapy. Due to the extensive tumor manifestation at actual presentation with pleural infiltrations and bilateral lung metastases, a surgical option was not considered. Systemic chemotherapy with doxorubicin and ifosfamide was initiated.

Alveolar soft-part sarcoma is an uncommon soft tissue tumor known for late metastases to lung, bone, and brain. Only Lillehei and coworkers reported an interval of 21 years between primary presentation and development of lung metastasis and 33 years between primary presentation and development of brain and renal masses [12].

To the best of our knowledge of the current literature, our case represents the second documented patient with such an extremely late tumor recurrence of alveolar soft-part sarcoma.
